# Reversine exhibits antineoplastic activity in JAK2^V617F^-positive myeloproliferative neoplasms

**DOI:** 10.1038/s41598-019-46163-2

**Published:** 2019-07-09

**Authors:** Keli Lima, Jorge Antonio Elias Godoy Carlos, Raquel de Melo Alves-Paiva, Hugo Passos Vicari, Fábio Pires de Souza Santos, Nelson Hamerschlak, Leticia Veras Costa-Lotufo, Fabiola Traina, João Agostinho Machado-Neto

**Affiliations:** 1 Department of Pharmacology, Biomedical Sciences Institute, University of São Paulo, São Paulo, Brazil; 2 Einstein’s Teaching and Research Institute, Albert Einstein Hospital, São Paulo, Brazil; 3 Department of Medical Images, Hematology and Clinical Oncology, University of São Paulo at Ribeirão Preto Medical School, Ribeirão Preto, São Paulo, Brazil

**Keywords:** Myeloproliferative disease, Myeloproliferative disease

## Abstract

JAK2/STAT signaling participates in the Ph-negative myeloproliferative neoplasms (MPN) pathophysiology and has been targeted by ruxolitinib, a JAK1/2 inhibitor. In the present study, the impact of ruxolitinib treatment on cytoskeleton-related genes expression was explored. In SET2 cells, AURKA and AURKB expression/activity were downregulated in a dose- and time-dependent manner by ruxolitinib. Reversine, a multikinase inhibitor selective for aurora kinases, reduced cell viability in a dose- and/or time-dependent manner in JAK2^V617F^ cells. Reversine significantly increased apoptosis and mitotic catastrophe, and reduced cell proliferation and clonogenic capacity in SET2 and HEL cells. In the molecular scenario, reversine induced DNA damage and apoptosis markers, as well as, reduced AURKA and AURKB expression/activity. In SET2 cells, reversine modulated the expression of 32 out of 84 apoptosis-related genes investigated, including downregulation of antiapoptotic (*BCL2*, *BCL2L1*, and *BIRC5*) and upregulation of proapoptotic (*BIK*, *BINP3*, and *BNIP3L*) genes. Synergism experiments indicated that low dose of reversine had a potentiating effect under ruxolitinib treatment at low doses in SET2 cells. In summary, our exploratory study establishes new targets, related to the regulation of the cellular cytoskeleton, for potential pharmacological intervention in MPN. These findings indicate that AURKA and AURKB participate in the JAK2/STAT signaling pathway and contribute to the MPN phenotype.

## Introduction

Philadelphia chromosome-negative myeloproliferative neoplasms (MPN) are characterized by excessive myeloid progenitor proliferation that keeps terminal differentiation capacity and have heightened risk of bone marrow failure or acute myeloid leukemia transformation^1^. The most frequent MPN are essential thrombocythemia (ET), polycythemia vera (PV), and primary myelofibrosis (PMF), which present a high incidence of JAK2 activating mutations (*i*.*e*. JAK2^V617F^, *indel* CALR, and MPL mutations)^2^. At the cellular level, the constitutive activation of the JAK2/STAT pathway leads to hypersensitive or independence of cytokines or growth factor, which contributes to cell proliferation, survival, and differentiation^3,4^. Thus, JAK2/STAT signaling pathway plays a central role in the MPN pathophysiology^5^ and has been targeted by ruxolitinib, a selective JAK1/2 inhibitor approved for the treatment of intermediate and high-risk PMF and PV patients that provides some clinical benefits, but does not lead to complete remission of these diseases and fails to eliminate the malignant clone^6–9^.

Previous studies from our research group have shown that the treatment with ruxolitinib modulates the activity of cytoskeleton-related proteins in JAK2^V617F^ cells^10^. Given that cellular cytoskeleton regulator proteins play vital functions and are often pharmacological targets in the treatment of several solid and hematological neoplasms^11^, the effects of ruxolitinib in this context deserve further exploration.

In the present study, we investigated the impact of the treatment with ruxolitinib on cytoskeleton-related genes expression and we identified aurora kinase A (AURKA) and B (AURKB) as promising targets for pharmacological intervention in MPN cellular models. The aurora kinase family comprises three serine/threonine kinases (AURKA, AURKB, and AURKC) that play an essential role in cell cycle progression during mitosis and cytokinesis, and their aberrant expression/activation have been related to the malignant phenotype of solid and hematological cancers^12–15^.

As a strategy for dual inhibition of AURKA and AURKB, reversine [2-(4-morpholinoanilino)-6-cyclohexylaminopurine] was employed. Reversine is a small synthetic purine analogue that induces mitotic catastrophe, cell-cycle arrest, polyploidy, and apoptosis in several cancer models^16–19^. Herein, we described the molecular and cellular effects of the treatment with reversine in the context of JAK2^V617F^-positive MPN.

## Results

### Pharmacological inhibition of JAK2/STAT signaling reduces AURKA and AURKB expression in JAK2^V617F^-positive cell line

**Figure 1:**
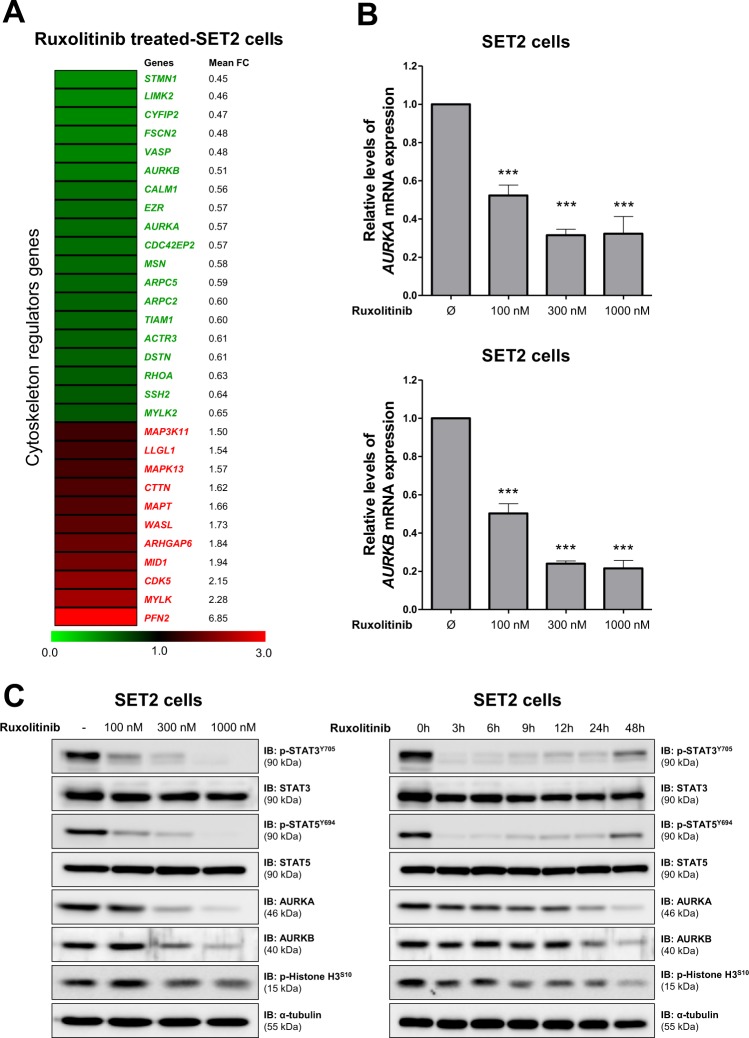
Pharmacological inhibition of JAK2/STAT signaling reduces AURKA and AURKB expression in SET2 cells. (**A**) Gene expression heatmap from RNA-seq analysis of SET2 cells upon the treatment with ruxolitinib. Data was obtained from Meyer *et al*.^20^ (GEO accession GSE69827). Gene expression was expressed as fold change of the mean of normalized counts of naïve SET2 cells, which was set as 1; genes demonstrating ≥1.5-fold in either direction are included in the heatmap. The mean of fold change obtained from three experimental replicates is indicated. (**B**) qPCR analysis of *AURKA* and *AURKB* mRNA expression in SET2 cells treated with graded concentrations of ruxolitinib (vehicle, 100, 300, or 1000 nM) for 48 hours. Bar graphs represent the mean ± SD of at least four independent experiments. The *p* values are indicated in the graphs; *p < 0.05, ***p < 0.0001; ANOVA test and Bonferroni post-test. (**C**) Western blot analysis for p-STAT3^Y705^, p-STAT5^Y694^, AURKA, AURKB, and p-histone H3^S10^, in total cell extracts from SET2 cells treated with graded concentrations of ruxolitinib (vehicle, 100, 300, or 1000 nM) for 48 hours (Right panel) or graded time of exposure (0, 3, 6, 9, 12, 24, and 48 hours) to ruxolitinib at 300 nM (Left panel); membranes were reprobed with the antibody for the detection of the respective total protein or α-tubulin, and developed with the SuperSignal™ West Dura Extended Duration Substrate system using a Gel Doc XR+ imaging system. Cropped blots are shown and full-length blots are included in Supplementary Fig. 7.

To initiate the exploratory study of the molecular effects of the treatment with ruxolitinib on cytoskeleton-related genes of JAK2^V617F^ positive cells, we first investigated the expression of 84 cytoskeleton-related genes in the RNA-Seq data from Meyer *et al*.^20^ (GSE69827). Using the cutoff of 1.5-fold for either direction, we found 30 genes differentially expressed (19 downregulated and 11 upregulated) in SET2 after the treatment with ruxolitinib for 48 hours (Fig. 1A). Since *AURKA* and *AURKB* gene expression has been associated with the activation of tyrosine kinase signaling in MPN, presented potential contribution to the malignant phenotype, and are putative druggable^21–23^, we selected these two genes for further validation. In SET2 cells, qPCR and Western blotting analysis confirmed that ruxolitinib reduces AURKA and AURKB expression in a dose- and time-dependent manner (Fig. 1B,C).

### Reversine induces apoptosis in SET2 and HEL cells

**Figure 2:**
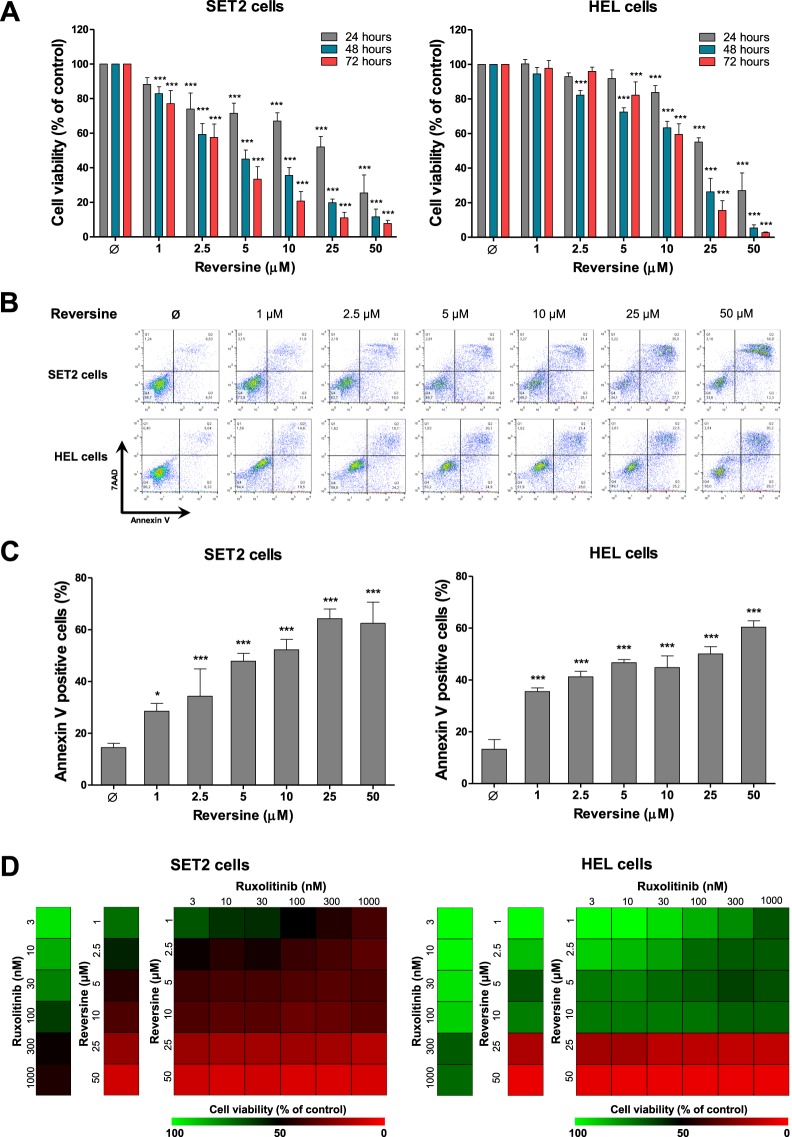
Reversine reduces cell viability of JAK2^V617F^-positive cell lines. (**A**) Dose- and time-response cytotoxicity were analyzed by methylthiazoletetrazolium (MTT) assay for SET2 and HEL cells treated with graded concentrations of reversine (1, 2.5, 5, 10, 25, and 50 μM) for 24, 48, and 72 hours. Values are expressed as the percentage of viable cells for each condition relative to untreated controls. Results are shown as the mean ± SD of at least four independent experiments. The *p* values and cell lines are indicated in the graphs; ***p < 0.0001; ANOVA test and Bonferroni post-test. (**B**) Apoptosis was detected by flow cytometry in SET2 and HEL cells treated with graded concentrations of reversine (1, 2.5, 5, 10, 25, and 50 μM) for 48 hours using an annexin V/7AAD staining method. Representative dot plots are shown for each condition; the upper and lower right quadrants (Q2 plus Q3) cumulatively contain the apoptotic population (annexin V+ cells). (**C**) Bar graphs represent the mean ± SD of at least four independent experiments quantifying apoptotic cell death. The *p* values and cell lines are indicated in the graphs; * *p* < 0.05, ***p < 0.0001; ANOVA test and Bonferroni post-test. (**D**) Dose-response cytotoxicity for combined treatment was analyzed by methylthiazoletetrazolium (MTT) assay for SET2 and HEL cells treated with graded concentrations of reversine (1, 2.5, 5, 10, 25, and 50 μM) and ruxolitinib (3, 10, 30, 100, 300, and 1000 nM) alone or in combination with each other for 48 hours. Values are expressed as the percentage of viable cells for each condition relative to untreated controls. Results are shown as the mean of at least three independent experiments.

Based on our findings that AURKA and AURKB were downregulated by JAK1/2 inhibitor, ruxolitinib, we investigated the effects of aurora kinases inhibition, by reversine, on cell viability in JAK2^V617F^-positive cell lines. The treatment with reversine reduced cell viability in a dose- and time-dependent manner (Fig. 2A). Using a nonlinear regression analysis, the IC_50_ values for reversine in SET2 were 23.6, 4.0, and 3.1 µM, and in HEL cells, they were 28.9, 13.4, and 11.8 µM at 24, 48 and 72 hours, respectively. In both JAK2^V617F^-positive cell lines analyzed, reversine strongly induces apoptosis in a dose-dependent manner (*p* < 0.05; Fig. 2B,C).

Next, we checked whether reversine-induced cytotoxicity could be affected by the treatment in combination with ruxolitinib. In SET2 cells, but not in HEL cells, low concentrations of reversine (2.5 μM) and ruxolitinib (<300 nM) combined showed potentiating effects in reducing cell viability (*p* < 0.001); Fig. 2D and Supplementary Fig. 1.

### Reversine decreases proliferation, clonogenicity, and cell cycle progression in JAK2^V617F^-positive cells

**Figure 3:**
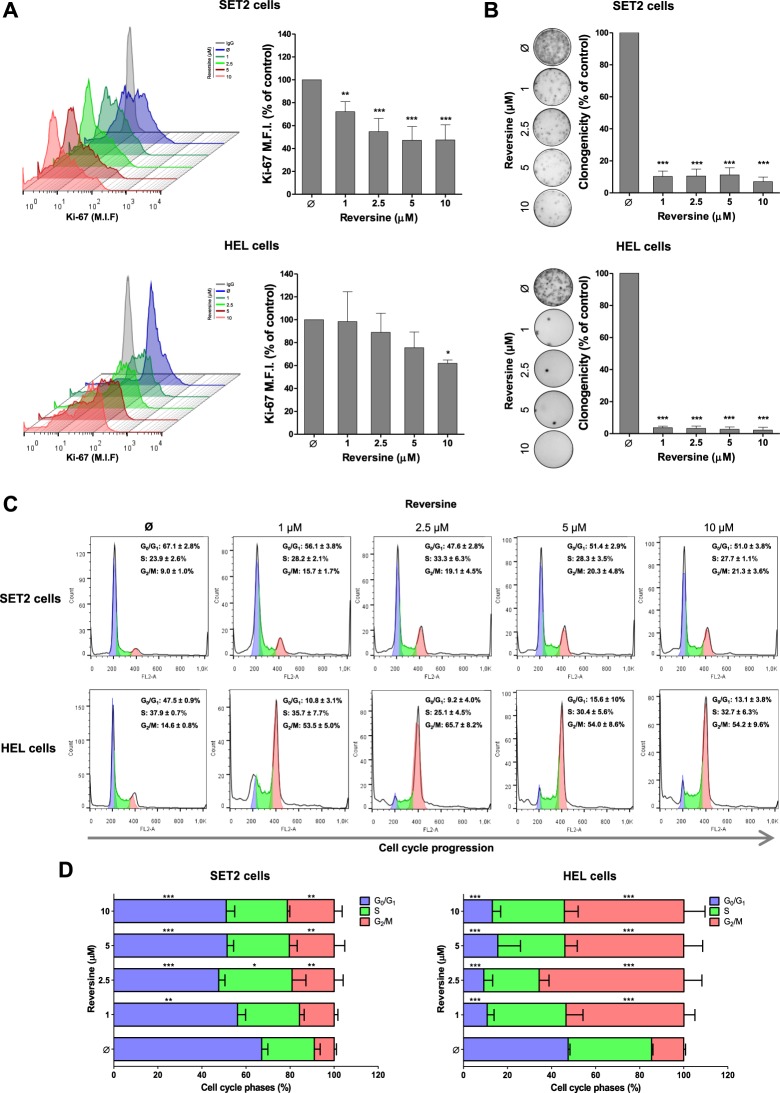
Reversine decreases proliferation, clonogenicity, and cell cycle progression in SET2 and HEL cells. (**A**) Ki-67 mean fluorescence intensity (M.F.I.) was determined by flow cytometry after incubation of SET2 or HEL cells treated with reversine (1, 2.5, 5, 10 μM) for 24 hours; histogram traces are illustrated. The bar graphs represent the Ki-67 M.F.I normalized to the respective untreated control cells, and results are shown as mean ± SD of at least four independent experiments. The *p* values and cell lines are indicated in the graphs; *p < 0.05, **p < 0.01, ***p < 0.001; ANOVA test and Bonferroni post-test. (**B**) Colonies containing viable cells were detected by MTT after 10 days of culture of SET2 and HEL cells treated reversine (1, 2.5, 5, 10 μM) for 36 hours and normalized to the corresponding DMSO-treated controls (Ø). Colony images are shown for one experiment and the bar graphs show the mean ± SD of at least four independent experiments. ***p < 0.0001; ANOVA test and Bonferroni post-test. (**C**) Cell cycle progression was determined by PI staining in SET2 or HEL cells treated with reversine (1, 2.5, 5, 10 μM) for 24 hours. A representative histogram for each condition is illustrated. (**D**) Bar graphs represent the mean ± SD of the percent of cells in G_0_/G_1_, S and G_2_/M phase upon reversine treatment of at the least four independent experiments. The *p* values and cell lines are indicated in the graphs; *p < 0.05, **p < 0.01, ***p < 0.001; ANOVA test and Bonferroni post-test.

Since aurora kinases are key proteins for cell cycle progression and success of cell division, we investigated the proliferation, clonogenicity, and cell cycle progression upon reversine exposure of JAK2^V617F^ cells. In SET2 and HEL cells, Ki-67 analysis revealed that short-time treatment with reversine significantly reduced cell proliferation (Fig. 3A) and autonomous clonal growth capacity (Fig. 3B), whereas long-term exposure to reversine completely inhibited colony formation in all concentration analyzed (Supplementary Fig. 2). The treatment with reversine significantly induces cell cycle arrest in G_2_/M phase in both JAK2^V617F^ cell lines (Fig. 3C,D).

### Reversine modulates apoptosis-related genes in JAK2^V617F^-positive cells

**Figure 4:**
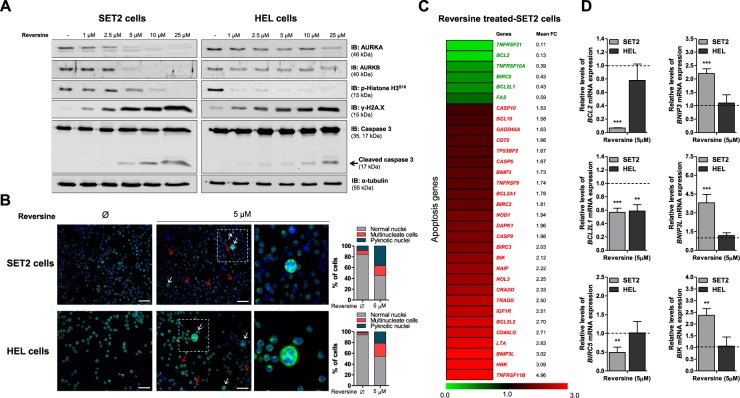
Reversine induces DNA damage and apoptosis markers in JAK2^V617F^-positive cell lines. (**A**) Western blot analysis for AURKA, AURKB, phospho(p)-histone H3^S10^, p-histone H2A.X^S139^ (**γ**-H2A.X) and Caspase 3 (total and cleaved) in total cell extracts from SET2 and HEL cells treated with graded concentrations of reversine (vehicle, 1, 2.5, 5, 10 or 25 μM) for 48 hours; membranes were reprobed with the antibody for the detection of the respective total protein or α-tubulin, and developed with the SuperSignal™ West Dura Extended Duration Substrate system using a Gel Doc XR+ imaging system. Cropped blots are shown and full-length blots are included in Supplementary Fig. 7. (**B**) Immunofluorescence analysis of SET2 and HEL cells treated or not with 5 μM reversine for 24 hours, displaying α-tubulin (green) and DAPI (blue) staining. Blank and red arrows illustrate mitotic catastrophe and nuclear fragmentation, respectively. Scale bars are shown in the figure (50 μm). Bar graphs represent the quantification of morphological findings of at least 200 cells for each condition. (**C**) Gene expression heatmap from qPCR array analysis of SET2 cells treated with reversine (5 μM). The mRNA levels are normalized to those of vehicle-treated SET2 cells and calculated as fold change in expression; genes demonstrating ≥1.5-fold in either direction compared to vehicle-treated cells upon the treatment with reversine are included in the heatmap. Two independent experiments of each condition were used for the analysis; green indicates repressed mRNA levels and red elevated mRNA levels. (**D**) qPCR analysis of *BCL2*, *BCL2L1*, *BIRC5*, *BNIP3*, *BNIP3L*, and *BIK* mRNA expression in SET2 and HEL cells treated with reversine (5 μM). The dashed line represents the mean gene expression in vehicle-treated cells and bars represent the fold change in gene expression in SET2 and HEL cells treated with reversine (5 μM) compared to their respective untreated cells. The *p* values and cell lines are indicated in the graphs. **p < 0.01, **p < 0.001; Student t test.

In the molecular scenario, reversine reduces AURK activity, as observed by the reduction in histone H3^S10^ phosphorylation, and increases markers of DNA damage (H2A.X phosphorylation) and apoptosis (cleaved caspase 3) (Fig. 4A). In SET2, but not in HEL cells, *AURKA* and *AURKB* mRNA downregulation upon the reversine was observed (Supplementary Fig. 3). Corroborating these data, morphological analysis indicates the presence of mitotic catastrophe and nuclear fragmentation (Fig. 4B).

In order to obtain additional insights about the molecular mechanisms involved in reversine-induced apoptosis, we investigated the expression of apoptosis-related genes in SET2 cells upon the treatment with reversine. A total of 32 different genes demonstrated a change in the expression of ≥1.5-fold in either direction compared to untreated cells: 6 downregulated genes (*TNFRSF21*, *BCL2*, *TNFRSF10A*, *BIRC5*, *BCL2L1*, and *FAS*) and 26 upregulated genes (*CASP10*, *BCL10*, *GADD45A*, *CD70*, *TP53BP2*, *CASP6*, *BNIP3*, *TNFRSF9*, *BCL2A1*, *BIRC2*, *NOD1*, *DAPK1*, *CASP9*, *BIRC3*, *BIK*, *NAIP*, *NOL3*, *CRADD*, *TRADD*, *IGF1R*, *BCL2L2*, *CD40LG*, *LTA*, *BNIP3L*, *HRK*, and *TNFRSF11B*) (Fig. 4C; Supplementary Table 1). Based on the cytotoxic effect previously observed, three downregulated-antiapoptotic genes (*BCL2*, *BCL2L1*, and *BIRC5*) and three upregulated-proapoptotic genes (*BNIP3*, *BNIP3L*, and *BIK*) were selected for validation in a larger number of experiments using SET2 and HEL cells, by qPCR. In SET2, but not in HEL cells, reduced *BCL2* and *BIRC5* expression and increased *BNIP3*, *BNIP3L*, and *BIK* expression upon the treatment with reversine were observed (all *p* < 0.01); *BCL2L1* expression was downregulated in both JAK2^V617F^ cell lines (*p* < 0.01, Fig. 4D).

## Discussion

Herein, we have investigated the effects of ruxolitinib on cytoskeleton-related genes in JAK2^V617F^-driven cellular models and a modulation of *AURKA* and *AURKB* was found. A previous study reported that JAK2^V617F^ mutant protein activates MYC/AURKA axis, which was identified to be critical for the malignant phenotype maintenance and chemotherapy resistance in transformed-Ba/F3 cells^22^. Similarly, Wen *et al*. reported that AURKA is upregulated in primary cells from MPN patients and that the genetic or pharmacological inhibition of AURKA reduces bone marrow fibrosis in JAK2^V617F^- and MPL^W515L^-driven models^23^. In STAT5B^N642H^-driven T cell neoplasia, AURKB is overactivated and represents potential therapeutic target^24^. Although the role of AURKA in JAK2^V617F^-mediated signaling is well described in the literature^22,23^, the functions of AURKB remain largely unknown in the context of MPN. Exploring microarray data containing gene expression profile of whole blood samples from MPN and healthy donors^25^ by GEOR2 (https://www.ncbi.nlm.nih.gov/geo/geo2r/), we found that both *AURKA* and *AURKB* are upregulated in PMF patients (GEO accession GSE26049; Supplementary Fig. 4), what corroborates previous studies^22,23^ and provides new insights for AURKB in MPN.

In the present study, AURKA and AURKB were pharmacologically targeted by reversine, a purine analogue that presents antineoplasic effects on multiple models of cancer^26^, including acute myeloid leukemia (AML)^27^, multiple myeloma^16^, and chronic myeloid leukemia (CML)^21^. Reversine reduced cell viability, proliferation, and cell cycle progression in JAK2^V617F^ cells. Exposure to reversine strongly inhibited autonomous growth, a characteristic that is usually associated with aggressiveness in hematological malignancies^28,29^, in SET2 and HEL cells. A modest potentiating effect was observed with the combination of low concentrations of reversine and ruxolitinib, corroborating the hypothesis that ruxolitinib inhibits AURK-mediated signaling in MPN models.

In JAK2^V617F^ cells, reversine reduced AURKA and AURKB expression/activity and induced DNA damage and apoptosis markers, which was similar to the one observed for AML and CML cell lines^21,27^. In SET2, but not in HEL cells, we observed downregulation of *BCL2* and *BIRC5* (antiapoptotic) and upregulation of *BNIP3*, *BNIP3L*, and *BIK* (proapoptotic) upon the treatment with reversine; *BCL2L1* (also known as BCL-XL) was downregulated in both JAK2^V617F^ cells tested. These findings pave avenues to identify unrecovered molecular effects of reversine on cancer cells. It is well defined that BCL2 family plays an important role in MPN: STAT proteins are transcription factors that induce *BCL2* and *BCL2L1* expression, which promotes cell survival^30–32^. Thus, the modulation of the BCL2 family after exposure to reversine may contribute to a key axis to determine the potency of the drug in JAK2^V617F^ cells. Indeed, some differences in the potency of reversine on cell viability, proliferation, and cell progression reduction between HEL and SET2 cells were observed. Of note, HEL cells are derived from an erythroleukemia patient^33^, featuring faster growth and resistant to JAK2 inhibitors *in vitro*, while SET2 cells are derived from an ET patient with leukemic transformation^34^, featuring slower growth and sensibility to JAK2 inhibitors *in vitro*. An interesting finding is that, in HEL cells, unlike what was observed in SET2 cells, inhibition of JAK2/STAT signaling by ruxolitinib did not strongly reduce the AURKA and AURKB expression/activation (Supplementary Fig. 5), which is consistent with failure to respond to ruxolitinib in cell viability assays. Thus, in the light of our results, further studies are needed to verify whether the AURK downregulation in the context of JAK2 inhibitors could be a predictor of therapeutic response in MPN patients. Despite differences in reversine potency between HEL and SET2, our findings support results from previous studies concluding that pharmacological inhibition of aurora kinases is a promising strategy in MPN^35,36^ and provides novel mechanistic insights.

Reversine is a multikinase inhibitor which, in addition to presenting selectivity for aurora kinases, has a high inhibitory potential for MPS1^37^ and JNK^38^. In view of the promising data of reversine in MPN cellular models, and the opportunity to identify new potential targets for these diseases, the effects of additional selective inhibitors for AURKA, AURKB, MPS1, and JNK were tested and all this inhibitor reduced cell viability with differences in their potencies (Supplementary Fig. 6) Of note, JNK, AURK, and MPS1 signaling pathways present cross-talk contributing to cell cycle progression. JNK acts upstream to AURKB^39^, whereas AURKB potentiates MPS1 activation^40^. Thus, our findings highlight that the multitarget effects of reversine may be attractive in diseases in which this signaling axis is activated.

In summary, our exploratory study establishes new targets related to regulation of cellular cytoskeleton for potential pharmacological intervention in MPN. In JAK2^V617F^ cells, the multikinase inhibition, including AURKA and AURKB, by reversine, may be a strategy to block cell cycle progression and reduce cell survival. Our findings corroborate the hypothesis that aurora kinases, particularly AURKA and AURKB, participate in the JAK2/STAT signaling pathway and contribute to the MPN phenotype.

## Material and Methods

### RNA-seq data analysis

RNA-seq data was obtained from Meyer *et al*.^20^, which was deposited in GEO database (https://www.ncbi.nlm.nih.gov/geo; GEO accession GSE69827). The expression of 84 cytoskeleton-related genes in samples from naïve SET2 cells (GSM1817344, GSM1817345, and GSM1817346) and ruxolitinib-treated SET2 cells (GSM1817332, GSM1817333, and GSM1817334) was investigated. Three replicates for each condition were available. Gene expression was expressed as fold change of the mean of normalized counts of naïve SET2 cells, which was set as 1. Genes that presented mean ≥1.5-fold either direction were included in the heatmap using multiple experiment viewer (MeV) 4.9.0 software (http://mev.tm4.org). The full list of genes included, normalized counts, and relative expression values are described in Supplementary Table 2.

### Cell culture and inhibitors

SET2 cells were kindly provided by Prof. Dr. Fabíola Attié de Castro (School of Pharmaceutical Sciences of Ribeirão Preto, University of São Paulo, Ribeirão Preto, Brazil). HEL was obtained from ATCC (Philadelphia, PA, USA). SET2 and HEL cells harboring JAK2^V617F^ mutation were tested and authenticated by Short Tandem Repeat (STR) matching analysis using the PowerPlex® 16 HS system (Promega, Madison, WI, USA) and the ABI 3500 Sequence Detector System (Life Technologies, Foster City, CA, USA). Cell culture conditions were performed in accordance with the recommendations of ATCC and DSMZ. All cell lines were mycoplasma-free. Ruxolitinib was obtained from InvivoGen (San Diego, CA, USA). Reversine was obtained from TargetMol (Target Molecule Corp., Boston, MA, USA). Aurora-A Inhibitor I (SML0882), AZD1152-HQPA (SML0268), NMS-P715 (475949) and SP600125 (S5567) were obtained from Sigma-Aldrich (St. Louis, MO, USA).

### Quantitative PCR (qPCR)

Total RNA was obtained using TRIzol reagent (Thermo Fisher Scientific). The cDNA was synthesized from 1 µg of RNA using High-Capacity cDNA Reverse Transcription Kit (Thermo Fisher Scientific). Quantitative PCR (qPCR) was performed with an ABI 7500 Sequence Detector System using the TaqMan system for *AURKA* (Hs00269212.m1), *AURKB* (Hs00177782.m1), *ATCB* (4310881E-1008027), and *GUS* (43108881E-0905024) genes (Life Technologies) or SyberGreen System for *AURKA*, *AURKB*, *BCL2*, *BCL2L1*, *BIRC5*, *BNIP3*, *BNIP3L*, and *BIK* (Supplementary Table 3). *ATCB*, *GUS*, and/or *HPRT1* were the reference genes. The relative quantification value was calculated using the equation 2^−ΔΔCT^. A negative ‘No Template Control’ was included for each primer pair.

### Western blot analysis

Equal amounts of protein were used as total extracts, followed by SDS-PAGE, Western blot analysis with the indicated antibodies and imaging using the SuperSignal™ West Dura Extended Duration Substrate System (Thermo Fisher Scientific, San Jose, CA, USA) and Gel Doc XR+ system (Bio-Rad, Hercules, CA, USA) or G:BOX Chemi XX6 gel doc systems (Syngene, Cambridge, UK). Antibodies against STAT3 (sc-7179), STAT5 (sc-835), AURKA (sc-25425), AURKB (sc-25426), phospho (p)-histone H3^S10^ (sc-8659-R), and p-histone H2A.X^S139^ (**γ**-H2A.X; sc-517348) were purchased from Santa Cruz Biotechnology (Santa Cruz, CA, USA). Antibodies against p-STAT3^Y705^ (#9131S), p-STAT5^Y694^ (#9359S), and caspase 3 (#9665) were obtained from Cell Signaling Technology (Danvers, MA, USA). Cropped gels retain important bands, but whole gel images are available in Supplementary Fig. 7.

### Cell viability assay

Cell viability was measured by methylthiazoletetrazolium (MTT) assay. SET2 (4 × 10^4^ cells/well) and HEL cells (2 × 10^4^ cells/well) were cultured in a 96-well plate in RPMI medium containing 10% or 20% FBS, respectively, in the presence of graded concentrations of reversine (1, 2.5, 5, 10, 25, and 50 μM) for 24, 48, and 72 hours. DMSO (Ø) was used as a negative control. IC_50_ values were calculated using a nonlinear regression analysis on GraphPad Prism 5 (GraphPad Software, Inc., San Diego, CA, USA). For combined treatment analysis, SET2 and HEL cells were treated with graded doses of reversine (1, 2.5, 5, 10, 25, and 50 μM) and ruxolitinib (3, 10, 30,100, 300, and 1000 nM) alone or in combination with each other for 48 hours and data were illustrated using multiple experiment viewer (MeV) 4.9.0 software (http://www.tm4.org/mev/).

### Apoptosis assay

SET2 and HEL were seeded in 24-well plates and treated with graded concentrations of reversine (1, 2.5, 5, 10, 25, and 50 μM) for 48 hours. Cells were then washed twice with ice cold PBS and resuspended in binding buffer containing 1 μg/mL 7AAD and 1 μg/mL FITC-labeled annexin V. All specimens were acquired by flow cytometry (FACSCalibur; Becton Dickinson) after incubation for 15 minutes at room temperature in a light-protected area and analyzed using FlowJo software (Treestar, Inc., San Carlos, CA, USA).

### Assessment of cell proliferation by Ki-67 staining

HEL and SET2 cells were treated with reversine (1, 2.5, 5, and 10 μM) for 24 hours, fixed with 70% ethanol and stored at −20 °C. Ki-67 staining was performed following the manufacturer’s instructions (Ki-67 FITC clone B56; BD Bioscience, San Jose, CA, USA) and the mean of fluorescence intensity (M.F.I.) was obtained by flow cytometry using a FACSCalibur instrument (Becton-Dickinson) and analyzed using FlowJo software (Treestar, Inc.). IgG isotype was used as negative control for each condition.

### Colony formation assay

Colony formation capacity was evaluated out in semisolid methylcellulose medium (2.5 × 10^3^ cells/mL for SET2 cells; 1 × 10^3^ cells/mL for HEL cells; MethoCult 4230; StemCell Technologies Inc., Vancouver, BC, Canada) after reversine exposure for 36 hours (equal number of viable cells were plated for each condition). Additionally, long-term exposure was also evaluated. Colonies were detected after 10 days of culture by adding 1 mg/mL of MTT reagent and scored by Image J quantification software (U.S. National Institutes of Health, Bethesda, MD, USA).

### Cell cycle analysis

SET2 and HEL cells were treated with reversine (1, 2.5, 5, and 10 μM) for 24 hours, fixed with 70% ethanol and stored at 4 °C for at least 2 hours. Then, fixed cells were stained with 20 μg/mL propidium iodide (PI) containing 10 μg/mL RNase A for 30 min at room temperature. DNA content distribution was acquired in a FACSCalibur cytometer (Becton-Dickinson) and analyzed using FlowJo software (Treestar, Inc.).

### Immunofluorescence microscopy

SET2 and HEL cells, treated or not with 5 μM reversine for 24 hours, were attached on cover slips coated with poly-L-lisine (1 mg/mL), fixed with 3.7% formaldehyde, permeabilized with 0.5% Triton X-PBS and blocked with 3% bovine serum albumin (BSA) PBS. Cells were then incubated with anti-α-tubulin Alexa Fluor® 488 conjugate (1:100 in 3% BSA PBS, Thermo Fisher Scientific) for 12 hours, and followed by PBS wash. The slides were mounted in ProLong Gold Anti-Fade Mounting Medium with DAPI (Thermo Fisher Scientific). Images were generated using fluorescence microscopy (Axio observer; Carl Zeiss, Welwyn Garden City, UK).

### PCR array profiling of apoptosis signaling-related genes

Total RNA from SET2 cells treated reversine (5 μM) for 24 hours was obtained using TRIzol reagent (Thermo Fisher Scientific). The cDNA was synthesized from 2 µg of RNA using RT2 First Strand Kit (Qiagen Sciences Inc., Germantown, MD, USA. PCR array was performed using the Human Apoptosis RT^2^ Profiler PCR Array kit (#PAHS-012ZA; Qiagen Sciences Inc.) according to the manufacturer’s instructions. The mRNA levels were normalized to those detected in untreated cells, and genes that presented a fold change ≥1.5-fold in any treatment were included in the heatmap using multiple experiment viewer (MeV) 4.9.0 software. Amplification was performed using an ABI 7500 Sequence Detector System (Thermo Fisher Scientific).

### Statistical analysis

Statistical analyses were performed using GraphPad Prism 5 (GraphPad Software, Inc.). For comparisons, ANOVA test and Bonferroni post-test or Student t test were used. A *p* value < 0.05 was considered as statistically significant. All pairs were analyzed and statistically significant differences are indicated.

## Supplementary information

Supplementary Tables and Figures
