# Supplementary material for: Reversine exhibits antineoplastic activity in JAK2V617F-positive myeloproliferative neoplasms
Source: Sci Rep. 2019 Jul 9;9:9895. doi: 10.1038/s41598-019-46163-2 (PMC6616334; doi:10.1038/s41598-019-46163-2)

Reversine exhibits antineoplastic activity in JAK2^{V617F}-positive myeloproliferative neoplasms

Keli Lima[1], Jorge Antonio Elias Godoy Carlos[1], Raquel de Melo Alves Paiva[2], Hugo Passos Vicari[1], Fábio Pires de Souza Santos[2], Nelson Hamerschlak[2], Leticia Veras Costa-Lotufo[1], Fabiola Traina[3], João Agostinho Machado-Neto[1]

[1]Department of Pharmacology, Biomedical Sciences Institute, University of São Paulo, São Paulo, Brazil

[2]Einstein's Teaching and Research Institute, Albert Einstein Hospital, São Paulo, Brazil

[3]Department of Medical Images, Hematology and Clinical Oncology, University of São Paulo at Ribeirão Preto Medical School, Ribeirão Preto, São Paulo, Brazil

Supplementary Material

Supplementary Table 1. Apoptosis-related genes investigated by PCR array.

Supplementary Table 2. RNA-seq data displayed as normalized counts and relative expression in naïve and ruxolitinib-treated SET2 cells.

Supplementary Table 3. Primer sequences and concentrations.

Supplementary Figure 1. Raw data of the synergism test in SET2 and HEL cells. Dose-response cytotoxicity for combined treatment were analyzed by methylthiazoletetrazolium (MTT) assay for SET2 and HEL cells treated with graded concentrations of reversine (1, 2.5, 5, 10, 25, and 50 μM) and ruxolitinib (3, 10, 30, 100, 300, and 1000 nM) alone or in combination with each other for 48 hours. Values are expressed as the percentage of viable cells for each condition relative to untreated controls. Results are shown as the mean±SD of four independent experiments.

Supplementary Figure 2. Long-term reversine exposure inhibits autonomous clonal growth in JAK2^{V617F} cell lines. Colonies containing viable cells were detected by MTT after 10 days of culture of SET2 and HEL cells exposed to reversine (1, 2.5, 5, 10 μM) and normalized to the corresponding DMSO-treated controls (Ø). Colony images are shown for one experiment and the bar graphs show the mean±SD of at least four independent experiments. ***p<0.0001; ANOVA test and Bonferroni post-test.

Supplementary Figure 3. Effects of reversine treatment on *AURKA* and *AURKB* mRNA expression in SET2 and HEL cells. qPCR analysis of *AURKA* and *AURKB* mRNA expression in SET2 **(A)** and HEL **(B)** cells treated with graded concentrations of reversine (vehicle, 1, 2.5, 5, or 10 μM) for 48 hours. Bar graphs represent the mean±SD of at least four independent experiments. The p values are indicated in the graphs; *p<0.05, **p<0.001, ***p<0.0001; ANOVA test and Bonferroni post-test.

Supplementary Figure 4. *AURKA* and *AURKB* are highly expressed in myeloproliferative neoplasms. Gene expression data was obtained from Skov *et al.*[25] (GEO accession GSE26049) for blood samples from polycythemia vera (PV), essential thrombocythemia (ET), and primary myelofibrosis (PMF) patients. The mean of *AURKA* (204092_s_at, 208079_s_at and 208080_at) and *AURKB* (209464_at and 239219_at) data obtained for each patient and number of patients are indicated; $*p<0.05$, $**p<0.01$, Kruskal-Wallis test and Dunn's post-test.

Supplementary Figure 5. Effects of ruxolitinib treatment on AURKA and AURKB expression/activation in HEL cells. qPCR analysis of *AURKA* **(A)** and *AURKB* **(B)** mRNA expression in HEL cells treated with graded concentrations of ruxolitinib (vehicle, 100, 300, or 1000 nM) for 48 hours. Bar graphs represent the mean±SD of at least four independent experiments. The *p* values are indicated in the graphs; $*p<0.05$, $**p<0.001$; ANOVA test and Bonferroni post-test. (**C**) Western blot analysis for p-$STAT3^{Y705}$, p-$STAT5^{Y694}$, AURKA, AURKB, and p-histone $H3^{S10}$, in total cell extracts from HEL cells treated with graded concentrations of ruxolitinib (vehicle, 100, 300, or 1000 nM) for 48 hours; membranes were reprobed with the antibody for the detection of the respective total protein or α-tubulin, and developed with the SuperSignal™ West Dura Extended Duration Substrate system using a Gel Doc XR+ imaging system.

Supplementary Figure 6. Cell viability upon treatment with AURKA, AURKB, MPS1, and JNK selective inhibitors in SET2 and HEL cells. Dose-response cytotoxicity was analyzed by methylthiazoletetrazolium (MTT) assay for SET2 and HEL cells treated with graded concentrations of Aurora-A Inhibitor I **(A)**, AZD1152-HQPA **(B)**, NMS-P715 **(C)** and SP600125 **(D)** for 48 hours. Values are expressed as the percentage of viable cells for each condition relative to untreated controls. Results are shown as the mean±SD of at least four independent experiments. The *p* values and cell lines are indicated in the graphs; $***p<0.0001$; ANOVA test and Bonferroni post-test.

Supplementary Figure 7. Whole gel images of western blotting analysis. Western blot analysis for protein phosphorylation and expression in total cell extracts from SET2 and HEL cells treated, or not, with ruxolitinib or reversine, as indicated; membranes were reprobed with the antibody for the detection of the respective total protein or α-tubulin. The molecular weight of the ladder, antibodies, merged and unmerged images are indicated.

Supplementary Table 1. Apoptosis-related genes investigated by PCR array.

Genes	Fold-change of vehicle-treated cells[1]
ABL1	1.2692
AIFM1	1.0683
AKT1	1.0043
APAF1	1.3342
BAD	1.3853
BAG1	1.1505
BAG3	0.9471
BAK1	0.8092
BAX	1.0409
BCL10	**1.583**
BCL2	**0.1287**
BCL2A1	**1.7841**
BCL2L1	**0.4319**
BCL2L10	1.4826
BCL2L11	1.3184
BCL2L2	**2.6974**
BFAR	1.1445
BID	0.7194
BIK	**2.124**
BIRC2	**1.8125**
BIRC3	**2.0317**
BIRC5	**0.4255**
BIRC6	1.3712
BNIP2	1.1957
BNIP3	**1.7342**
BNIP3L	**3.0151**
BRAF	0.9937
CASP1	0.7231
CASP10	**1.5275**
CASP14	1.4826
CASP2	0.7651
CASP3	1.3006
CASP4	1.3117
CASP5	1.0127
CASP6	**1.6737**
CASP7	0.7542
CASP8	1.2773
CASP9	**1.983**
CD27	0.8254
CD40	0.7837
CD40LG	**2.7122**
CD70	**1.6577**
CFLAR	1.1613
CIDEA	1.4826
CIDEB	0.9143
CRADD	**2.3315**
CYCS	0.8066

DAPK1	**1.9623**
DFFA	0.8882
DIABLO	0.9912
FADD	0.7107
FAS	**0.5849**
FASLG	0.9095
GADD45A	**1.6322**
HRK	**3.0879**
IGF1R	**2.5088**
IL10	1.4826
LTA	**2.832**
LTBR	0.9763
MCL1	0.8694
NAIP	**2.2244**
NFKB1	1.2184
NOD1	**1.9416**
NOL3	**2.2481**
PYCARD	1.0145
RIPK2	0.9574
TNF	1.466
TNFRSF10A	**0.3912**
TNFRSF10B	0.8018
TNFRSF11B	**4.9584**
TNFRSF1A	1.2312
TNFRSF1B	0.8008
TNFRSF21	**0.1123**
TNFRSF25	1.2506
TNFRSF9	**1.7421**
TNFSF10	0.7602
TNFSF8	1.4826
TP53	1.13
TP53BP2	**1.6706**
TP73	1.4826
TRADD	**2.4965**
TRAF2	1.1254
TRAF3	0.7729
XIAP	1.1235

[1]SET2 were treated with 5 μM reversine. The data represent the mean of two independent experiments for each condition.

Relative gene expression that presents 1.5 fold-change of control is highlighted in bold.

Supplementary Table 2. RNA-seq data displayed as normalized counts and relative expression in naïve and ruxolitinib-treated SET2 cells.

Genes	Normalized counts[1]						Relative expression					
	Naïve SET2 cells			Ruxolitinib-treated SET2 cells			Naïve SET2 cells			Ruxolitinib-treated SET2 cells		
	#1	#2	#3	#1	#2	#3	#1	#2	#3	#1	#2	#3
ACTR2	18148.526	13981.960	14472.537	11084.293	11448.082	10316.582	1.17	0.90	0.93	0.71	0.74	0.66
ACTR3	9258.280	7380.776	7391.685	4740.500	5060.644	4929.490	1.16	0.92	0.92	0.59	0.63	0.62
ARAP1	4809.055	7427.141	7204.166	8341.817	8557.672	8783.564	0.74	1.15	1.11	1.29	1.32	1.36
ARFIP2	1121.683	1345.684	1467.534	1187.938	1141.411	1333.756	0.86	1.03	1.12	0.91	0.87	1.02
ARHGAP6	511.028	484.144	486.121	833.525	1003.588	890.172	1.03	0.98	0.98	1.69	2.03	1.80
ARHGDIB	30355.766	24871.435	23992.147	18999.502	20650.224	21519.332	1.15	0.94	0.91	0.72	0.78	0.81
ARHGEF11	1015.023	1472.920	1584.733	1247.006	1261.764	1252.649	0.75	1.08	1.17	0.92	0.93	0.92
ARPC1B	2945.443	3177.669	3267.302	4270.763	3606.704	4253.600	0.94	1.02	1.04	1.36	1.15	1.36
ARPC2	8213.955	8703.816	8680.873	5114.601	5181.968	5014.602	0.96	1.02	1.02	0.60	0.61	0.59
ARPC3	6828.553	3128.069	2670.097	2690.909	2926.322	3042.005	1.62	0.74	0.63	0.64	0.70	0.72
ARPC4	2538.731	2407.783	1987.286	2211.796	2153.734	2559.370	1.10	1.04	0.86	0.96	0.93	1.11
ARPC5	2583.270	2377.591	2203.340	1473.905	1348.146	1404.850	1.08	1.00	0.92	0.62	0.56	0.59
AURKA	2395.737	1897.760	1788.557	1108.242	1090.941	1273.677	1.18	0.94	0.88	0.55	0.54	0.63
AURKB	2361.746	2384.061	2510.095	1245.131	1150.146	1271.674	0.98	0.99	1.04	0.51	0.48	0.53
AURKC	12.893	21.565	41.784	22.502	26.206	16.021	0.51	0.85	1.64	0.89	1.03	0.63
BAIAP2	267.235	357.986	295.545	248.464	268.853	345.455	0.87	1.17	0.96	0.81	0.88	1.13
CALD1	1.172	17.252	0.000	0.000	3.882	10.013	0.19	2.81	0.00	0.00	0.63	1.63
CALM1	5250.930	3935.695	3713.677	2297.117	2374.057	2525.325	1.22	0.92	0.86	0.53	0.55	0.59
CASK	264.891	219.968	252.742	176.269	244.588	178.235	1.08	0.89	1.03	0.72	0.99	0.72
CCNA1	112.520	91.653	79.491	68.445	93.176	90.119	1.19	0.97	0.84	0.72	0.99	0.95
CCNB2	1616.302	1583.982	1367.660	1225.442	1285.058	1352.781	1.06	1.04	0.90	0.80	0.84	0.89
CDC42	9769.308	7331.175	8480.106	6072.827	6078.791	5945.828	1.15	0.86	0.99	0.71	0.71	0.70
CDC42BPA	6774.637	6249.668	6446.959	6520.062	6987.261	6460.506	1.04	0.96	0.99	1.00	1.08	1.00
CDC42EP2	117.208	171.445	154.906	64.694	96.088	93.123	0.79	1.16	1.05	0.44	0.65	0.63
CDC42EP3	918.913	848.601	864.215	669.446	702.705	744.981	1.05	0.97	0.99	0.76	0.80	0.85
CDK5	110.176	133.706	81.530	273.779	192.176	232.306	1.02	1.23	0.75	2.52	1.77	2.14
CDK5R1	50.400	48.522	52.994	65.632	69.882	52.069	1.00	0.96	1.05	1.30	1.38	1.03
CFL1	20193.810	17172.569	16592.309	16201.707	15484.756	17938.617	1.12	0.95	0.92	0.90	0.86	1.00
CIT	4744.590	5344.997	5478.794	3650.072	3606.704	3394.469	0.91	1.03	1.06	0.70	0.70	0.65

CLASP1	4704.739	4179.384	4535.088	4315.767	4679.203	4574.022	1.05	0.93	1.01	0.96	1.05	1.02
CLASP2	2088.651	1697.201	1819.131	1985.834	2151.793	2047.696	1.12	0.91	0.97	1.06	1.15	1.10
CLIP1	2008.949	1555.947	1568.427	1292.011	1475.293	1272.676	1.17	0.91	0.92	0.76	0.86	0.74
CLIP2	2191.794	2090.770	2171.747	1847.070	1827.617	1953.572	1.02	0.97	1.01	0.86	0.85	0.91
CRK	1477.996	1303.632	1105.746	1034.171	1190.911	1165.534	1.14	1.01	0.85	0.80	0.92	0.90
CTTN	282.472	486.301	495.293	618.815	631.853	798.051	0.67	1.15	1.18	1.47	1.50	1.89
CYFIP1	6499.198	6537.567	6443.902	7202.634	6946.496	7295.605	1.00	1.01	0.99	1.11	1.07	1.12
CYFIP2	1358.444	969.367	1069.058	531.619	543.529	526.693	1.20	0.86	0.94	0.47	0.48	0.47
DIAPH1	20533.714	16486.787	17386.204	14331.198	14734.492	14211.711	1.13	0.91	0.96	0.79	0.81	0.78
DSTN	3095.470	2185.658	1872.125	1478.593	1438.411	1468.934	1.30	0.92	0.79	0.62	0.60	0.62
EZR	5724.451	4732.538	4117.249	2779.980	2692.410	2796.682	1.18	0.97	0.85	0.57	0.55	0.58
FNBP1L	722.003	591.972	618.606	570.060	582.353	577.761	1.12	0.92	0.96	0.88	0.90	0.90
FSCN2	0.000	0.000	2.038	0.000	0.971	0.000	0.00	0.00	3.00	0.00	1.43	0.00
GSN	1262.333	1153.752	1359.507	1421.400	1652.911	1617.129	1.00	0.92	1.08	1.13	1.31	1.28
IQGAP1	9408.307	7129.538	7298.945	5608.716	5544.967	5511.256	1.18	0.90	0.92	0.71	0.70	0.69
IQGAP2	2573.893	2057.344	2013.783	1574.228	1560.705	1470.937	1.16	0.93	0.91	0.71	0.70	0.66
LIMK1	1889.397	2042.248	1880.278	2148.039	2015.911	2115.786	0.98	1.05	0.97	1.11	1.04	1.09
LIMK2	3331.059	3505.464	3655.587	1592.980	1552.940	1725.271	0.95	1.00	1.05	0.46	0.44	0.49
LLGL1	826.318	1207.665	1203.582	1696.116	1662.617	1614.125	0.77	1.12	1.12	1.57	1.54	1.50
MACF1	29796.683	31017.589	32572.126	21336.936	23092.222	19989.317	0.96	1.00	1.05	0.69	0.74	0.64
MAP3K11	2728.608	3697.397	3358.004	4999.277	4604.468	5034.628	0.84	1.13	1.03	1.53	1.41	1.54
MAP4	4946.188	4806.939	5163.886	5468.076	5464.409	5215.867	0.99	0.97	1.04	1.10	1.10	1.05
MAPK13	1.172	40.974	43.822	39.379	36.882	59.078	0.04	1.43	1.53	1.37	1.29	2.06
MAPRE1	6769.949	5191.882	4908.087	4064.491	4799.556	4447.856	1.20	0.92	0.87	0.72	0.85	0.79
MAPRE2	2335.961	2197.519	2269.582	2499.638	2719.587	2602.426	1.03	0.97	1.00	1.10	1.20	1.15
MAPT	10.549	4.313	1.019	7.501	5.824	13.017	1.99	0.81	0.19	1.42	1.10	2.46
MARK2	988.066	1206.587	1201.544	1619.233	1488.882	1599.105	0.87	1.07	1.06	1.43	1.32	1.41
MID1	17.581	32.348	12.229	27.190	21.353	72.095	0.85	1.56	0.59	1.31	1.03	3.48
MSN	67741.683	56741.937	59758.198	35434.671	35667.157	35881.240	1.10	0.92	0.97	0.58	0.58	0.58
MYLK	956.419	819.487	789.819	1853.633	1875.175	2119.791	1.12	0.96	0.92	2.17	2.19	2.48
MYLK2	11.721	15.096	12.229	7.501	8.735	9.012	0.90	1.16	0.94	0.58	0.67	0.69
NCK1	1127.543	787.139	668.543	755.705	775.500	767.010	1.31	0.91	0.78	0.88	0.90	0.89
NCK2	283.644	278.194	243.570	323.472	325.147	289.381	1.06	1.04	0.91	1.20	1.21	1.08

PAK1	553.223	539.136	511.599	281.280	395.029	431.568	1.03	1.01	0.96	0.53	0.74	0.81
PAK4	1031.433	1228.152	1371.737	1442.965	1425.793	1423.875	0.85	1.01	1.13	1.19	1.18	1.18
PFN2	5.860	8.626	7.134	13.126	9.706	125.165	0.81	1.20	0.99	1.82	1.35	17.37
PHLDB2	5.860	22.644	1.019	0.000	0.000	22.029	0.60	2.30	0.10	0.00	0.00	2.24
PIKFYVE	5562.704	5547.712	5926.189	4751.751	4914.085	4629.094	0.98	0.98	1.04	0.84	0.87	0.82
PPP1R12A	4534.787	3794.441	3862.469	3917.288	4190.027	3946.195	1.12	0.93	0.95	0.96	1.03	0.97
PPP1R12B	915.396	1244.327	1305.494	1562.977	1697.558	1609.119	0.79	1.08	1.13	1.35	1.47	1.39
PPP3CA	2619.605	2209.380	2029.070	2094.596	2259.528	2186.879	1.15	0.97	0.89	0.92	0.99	0.96
PPP3CB	1006.819	767.730	742.939	757.580	790.058	669.882	1.20	0.91	0.89	0.90	0.94	0.80
RAC1	4237.078	3257.461	3090.994	3019.068	3046.675	3411.491	1.20	0.92	0.88	0.86	0.86	0.97
RACGAP1	2871.602	2299.955	2522.324	1926.766	2027.558	2001.635	1.12	0.90	0.98	0.75	0.79	0.78
RDX	5327.115	3405.185	3574.057	3187.836	3226.233	2931.860	1.30	0.83	0.87	0.78	0.79	0.71
RHOA	28074.893	20387.978	21751.099	14332.135	14816.021	14774.451	1.20	0.87	0.93	0.61	0.63	0.63
ROCK1	3976.876	2773.317	2954.432	2585.897	2680.763	2470.252	1.23	0.86	0.91	0.80	0.83	0.76
SSH1	1243.580	1434.103	1477.725	1554.539	1745.117	1670.199	0.90	1.04	1.07	1.12	1.26	1.21
SSH2	3533.829	3280.105	3362.080	2198.669	2293.499	2029.672	1.04	0.97	0.99	0.65	0.68	0.60
STMN1	24279.690	16995.732	16888.873	8606.219	9074.995	8410.072	1.25	0.88	0.87	0.44	0.47	0.43
TIAM1	2815.342	3340.488	3388.577	1894.887	2012.999	1833.414	0.88	1.05	1.07	0.60	0.63	0.58
VASP	30496.416	25776.106	28563.923	12725.091	13897.845	13924.332	1.08	0.91	1.01	0.45	0.49	0.49
WAS	2877.463	3036.416	3003.350	2342.122	2287.675	2502.294	0.97	1.02	1.01	0.79	0.77	0.84
WASF1	856.792	866.931	832.622	692.886	837.617	794.045	1.01	1.02	0.98	0.81	0.98	0.93
WASL	144.166	174.680	163.059	328.160	259.147	246.324	0.90	1.09	1.02	2.04	1.61	1.53

[1]RNA-seq data was obtained from GEO database (https://www.ncbi.nlm.nih.gov/geo; GEO accession GSE69827; naïve SET2 cells [GSM1817344. GSM1817345 and GSM1817346] and ruxolitinib-treated SET2 cells [GSM1817332. GSM1817333 and GSM1817334].

Supplementary Table 3. Primer sequences and concentrations.

Gene	Sequence	Concentration
AURKA	FW: CCACCTTCGGCATCCTAATA<br>RV: TCCAAGTGGTGCATATTCCA	300 nM
AURKB	FW: CCCTGAGGAGGAAGACAATG<br>RV: GCACCACAGATCCACCTTCT	300 nM
BCL2	FW: ATGTGTGTGGAGAGCGTCAA<br>RV: ACAGTTCCACAAAGGCATCC	300 nM
BCL2L1	FW: CTTGGATGGCCACTTACCTGAA<br>RV: GCTGCTGCATTGTTCCCATA	300 nM
BIRC5	FW: GCCCAGTGTTTCTTCTGCTTCA<br>RV: GCACTTTCTCCGCAGTTTCCTC	300 nM
BNIP3	FW: ATATGGGATTGGTCAAGTCGG<br>RV: CGCTCGTGTTCCTCATGCT	300 nM
BNIP3L	FW: ACACCAGCAGGGACCATAGC<br>RV: TTTCTTCAAAGCCTCGACTTCC	300 nM
BIK	FW: TCTGAAGAGGACCTGGACCCT<br>RV: GGCTCACGTCCATCTCGTC	300 nM
HPRT1	FW: GAACGTCTTGCTCGAGATGTGA<br>RV: TCCAGCAGGTCAGCAAAGAAT	150 nM
ACTB	FW: AGGCCAACCGCGAGAAG<br>RV: ACAGCCTGGATAGCAACGTACA	150 nM

Supplementary Figure 1

SET2 cells

Ruxolitinib (nM)	
3	95.3±1.1
10	83.8±5.9
30	75.9±3.6
100	62.5±5.4
300	48.1±2.6
1000	44.2±2.0

Reversine (µM)	
1	71.5±5.9
2.5	57.1±10
5	41.6±5.2
10	35.3±4.9
25	20.8±3.1
50	8.7±3.2

Reversine (µM) \ Ruxolitinib (nM)	3	10	30	100	300	1000
1	67.4±9.6	59.9±10	59.3±6.6	50.3±8.5	42.7±6.6	36.3±5.7
2.5	47.5±5.6	42.3±3.7	45.8±5.7	38.6±5.7	35.9±5.1	32.5±6.4
5	37.5±2.8	35.5±5.3	35.7±4.7	34.1±4.4	32.6±4.8	34.5±4.6
10	35.2±2.7	31.5±6.1	33.0±5.1	28.8±7.4	30.4±6.4	33.0±2.7
25	19.9±1.9	17.6±4.2	18.2±1.3	19.4±2.9	18.3±1.9	14.2±7.8
50	9.8±1.3	7.8±2.1	8.7±1.9	6.9±3.4	7.2±2.8	7.8±2.5

HEL cells

Ruxolitinib (nM)	
3	100±14
10	98.7±19
30	95.1±9.7
100	91.4±12
300	67.6±16
1000	71.4±9.4

Reversine (µM)	
1	100±13
2.5	88.2±17
5	66.8±12
10	74.7±12
25	15.9±5.2
50	4.4±1.2

Reversine (µM) \ Ruxolitinib (nM)	3	10	30	100	300	1000
1	101.3±17	99.6±18	93.9±16	85.1±8.5	78.4±10	67.0±5.5
2.5	91.1±5.5	87.0±5,5	81.8±8.9	70.7±6.1	68.2±5.9	68.3±2.5
5	74.0±5.8	76.3±6.9	71.2±4.5	68.1±5.1	63.2±7.6	65.6±10
10	74.2±4.4	74.4±4.9	73.6±4.3	73.0±8.9	69.5±12	68.8±7.8
25	17.7±7.1	17.7±2.8	12.8±2.6	13.0±1.7	12.0±2.6	12.5±2.8
50	4.9±1.9	4.3±1.6	4.0±0.8	4.0±0.6	4.4±0.7	4.0±0.7

Supplementary Figure 2

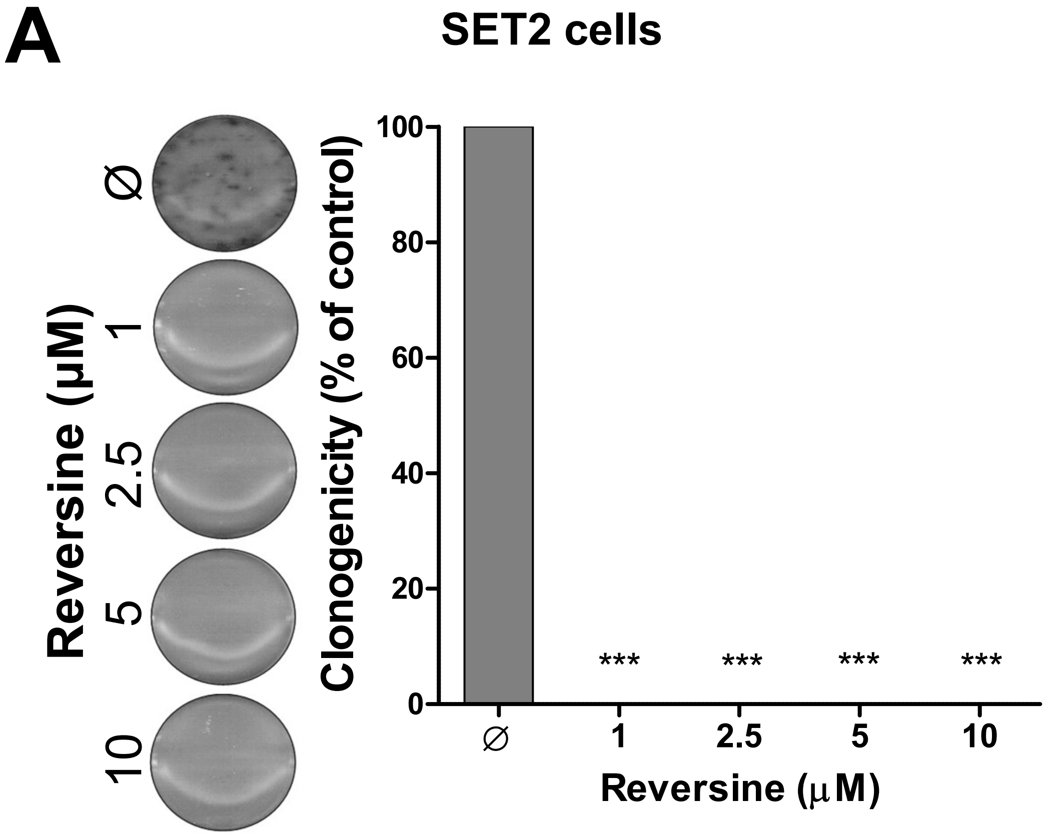

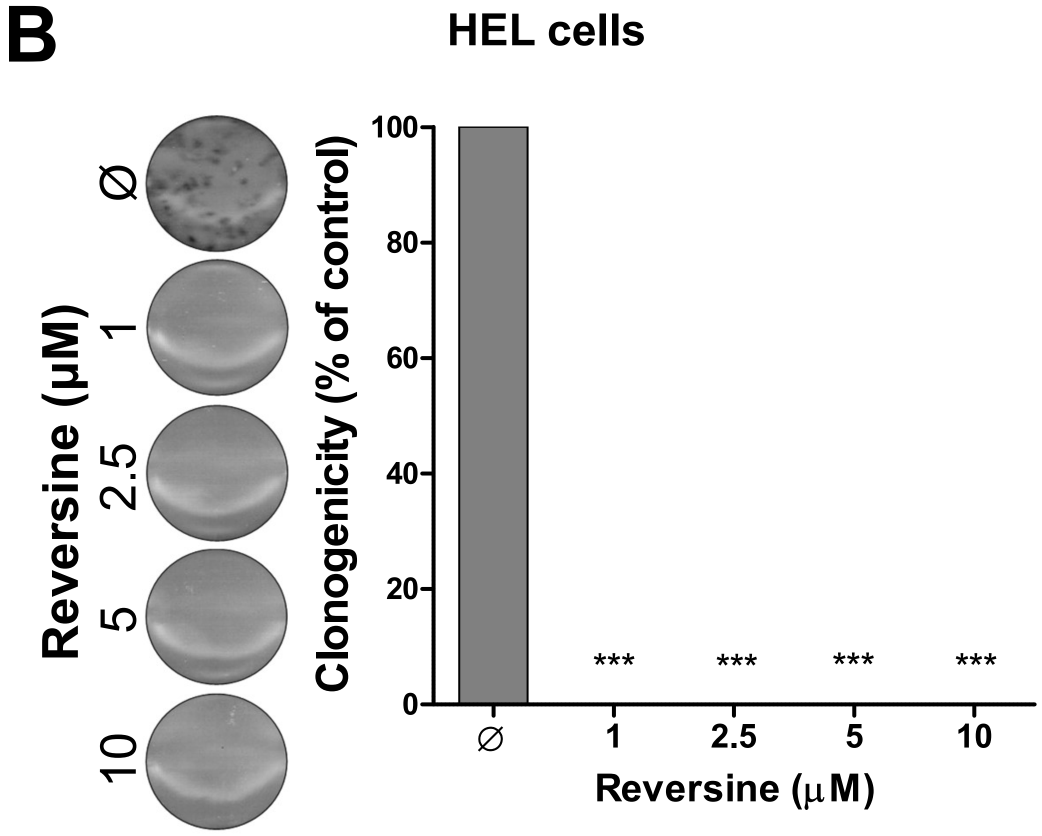

Supplementary Figure 3

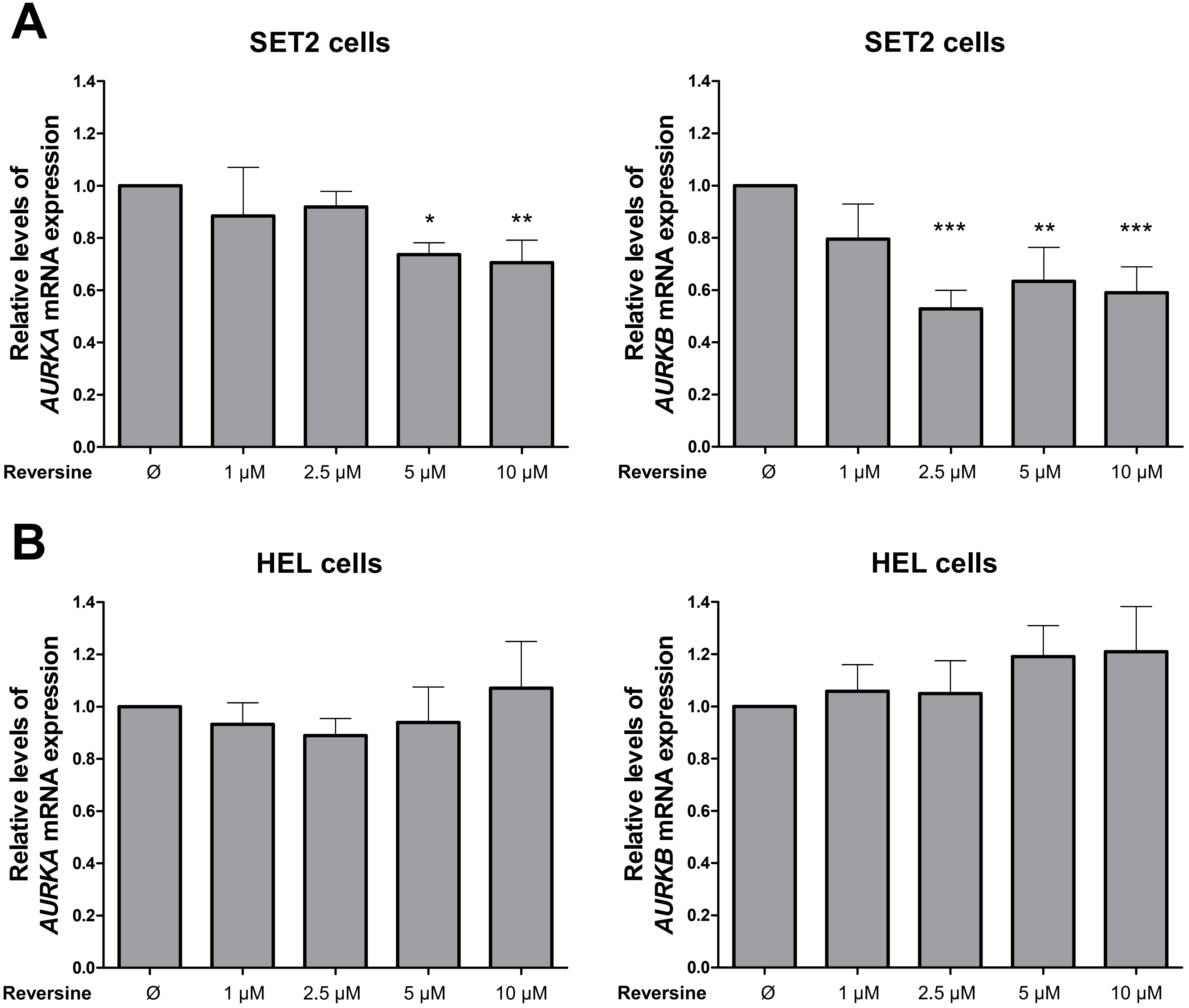

Suplementary Figure 4

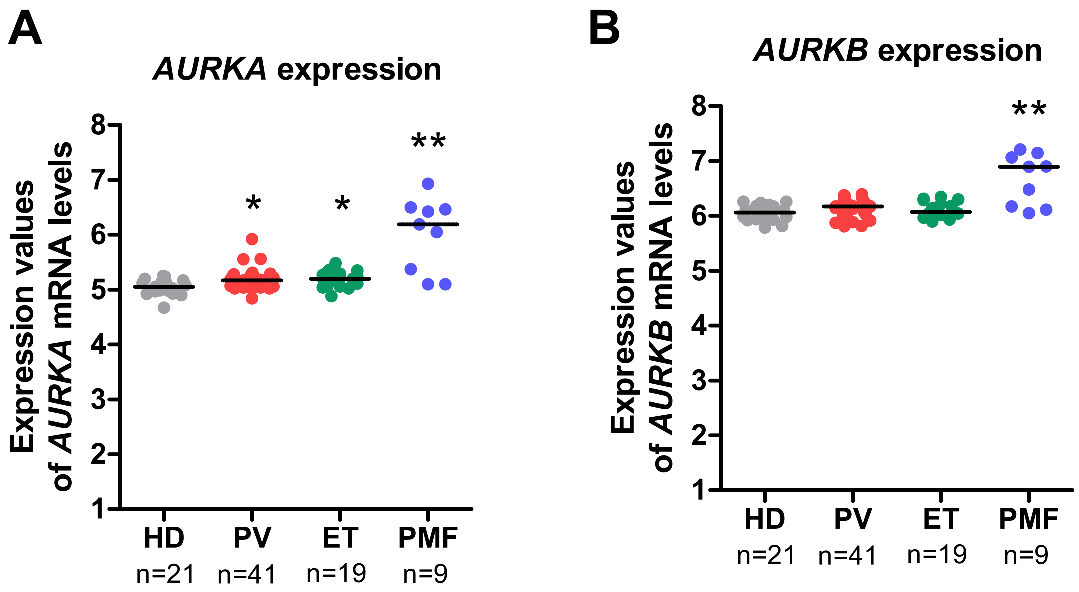

Supplementary Figure 5

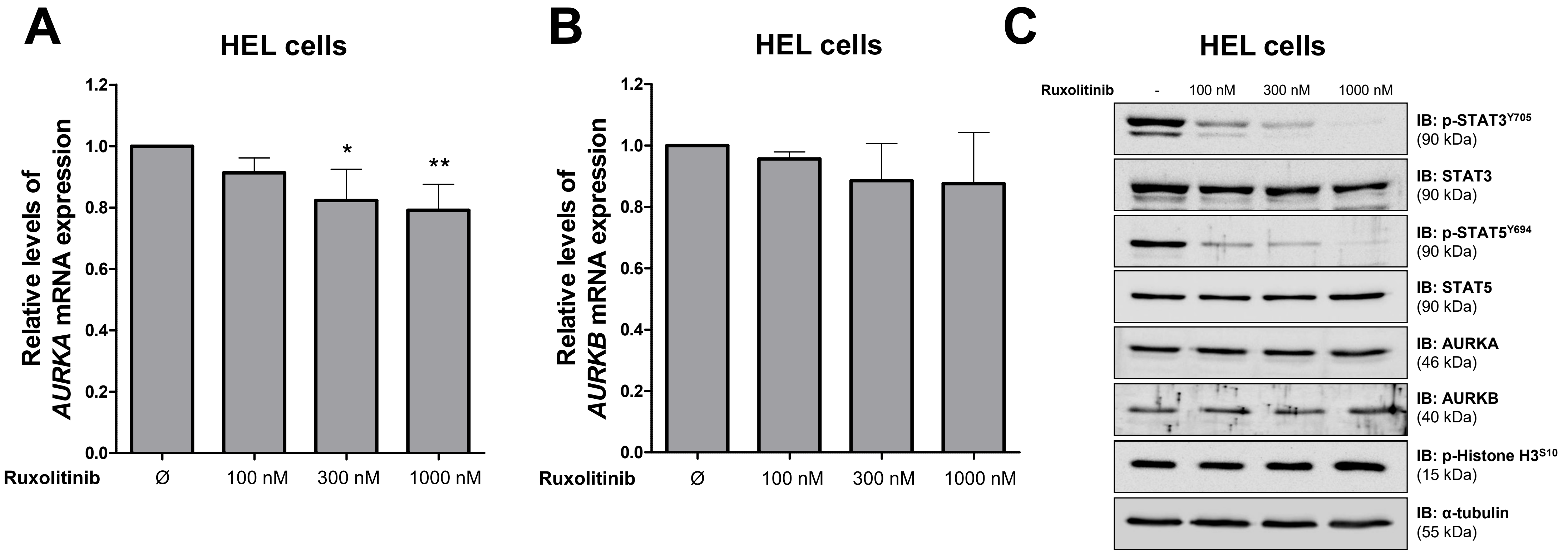

Supplementary Figure 6

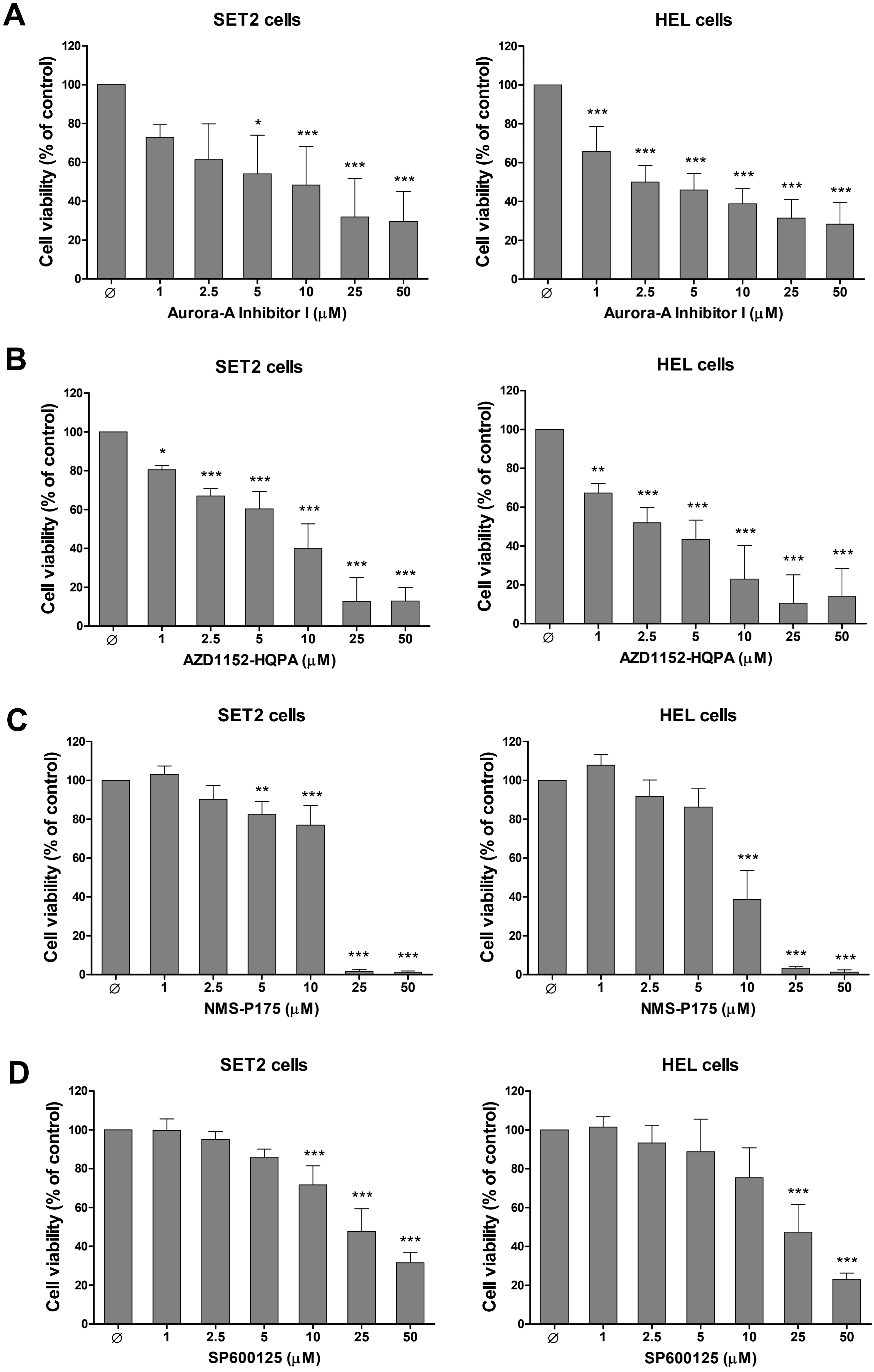

Supplementary Figure 7

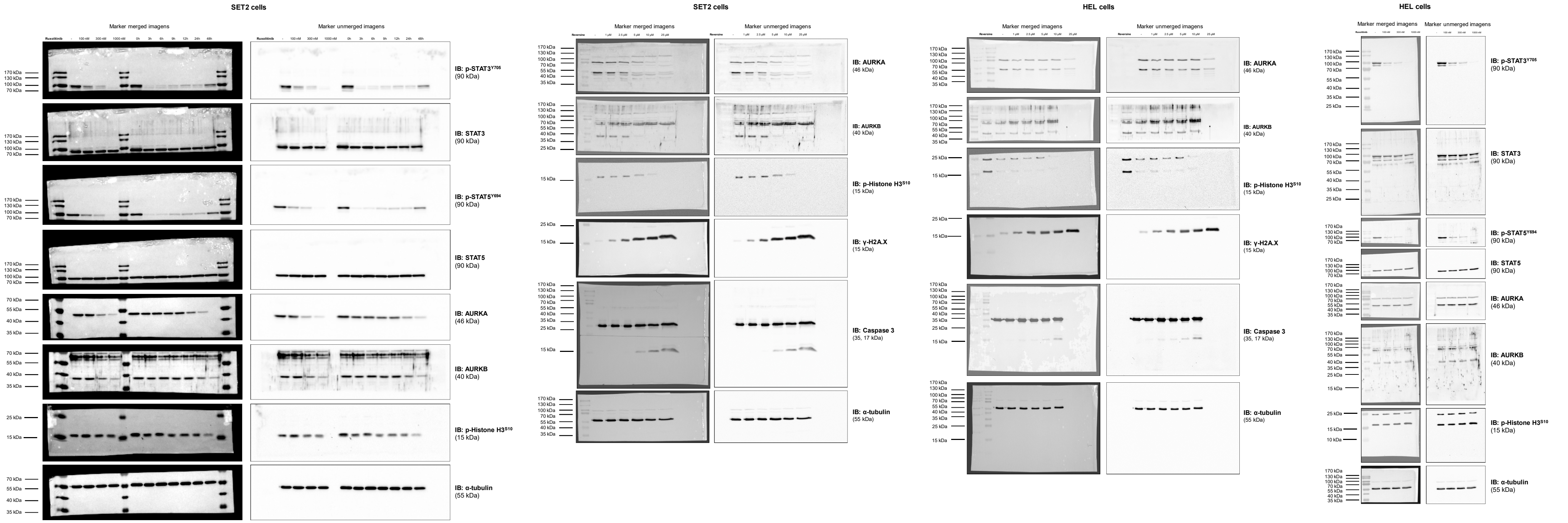

Supplement: Supplementary file 1 — Supplementary Tables and Figures [file 41598_2019_46163_MOESM1_ESM.pdf]
